# A Synthetic Small RNA Homologous to the D-Loop Transcript of mtDNA Enhances Mitochondrial Bioenergetics

**DOI:** 10.3389/fphys.2022.772313

**Published:** 2022-04-06

**Authors:** Theodore L. Mathuram, Danyelle M. Townsend, Vincent J. Lynch, Ilya Bederman, Zhi-Wei Ye, Jie Zhang, Wade J. Sigurdson, Erin Prendergast, Raul Jobava, Jonathan P. Ferruzza, Mary R. D’Angelo, Maria Hatzoglou, Yaron Perry, Anna Blumental-Perry

**Affiliations:** ^1^Department of Biochemistry, Jacobs School of Medicine and Biomedical Sciences, University at Buffalo, State University of New York, Buffalo, NY, United States; ^2^Department of Drug Discovery & Biomedical Sciences, College of Pharmacy, Medical University of South Carolina, Charleston, SC, United States; ^3^Department of Biological Sciences, College of Arts and Sciences, University at Buffalo, State University of New York, Buffalo, NY, United States; ^4^Department of Genetics and Genome Sciences, School of Medicine, Case Western Reserve University, Cleveland, OH, United States; ^5^Department of Medicine, Confocal Microscope and Flow Cytometry Facility, Jacobs School of Medicine and Biomedical Sciences, University at Buffalo, State University of New York, Buffalo, NY, United States; ^6^Division of Thoracic Surgery, Department of Surgery, Jacobs School of Medicine and Biomedical Sciences, University at Buffalo, State University of New York, Buffalo, NY, United States

**Keywords:** mitochondria, mitochondria-to-nucleus retrograde signaling, small ncRNA, Krebs cycle, OxPhos, D-loop transcripts

## Abstract

Mitochondrial malfunction is a hallmark of many diseases, including neurodegenerative disorders, cardiovascular and lung diseases, and cancers. We previously found that alveolar progenitor cells, which are more resistant to cigarette smoke-induced injury than the other cells of the lung parenchyma, upregulate the mtDNA-encoded small non-coding RNA mito-ncR-805 after exposure to smoke. The mito-ncR-805 acts as a retrograde signal between the mitochondria and the nucleus. Here, we identified a region of mito-ncR-805 that is conserved in the mammalian mitochondrial genomes and generated shorter versions of mouse and human transcripts (mmu-CR805 and hsa-LDL1, respectively), which differ in a few nucleotides and which we refer to as the “functional bit”. Overexpression of mouse and human functional bits in either the mouse or the human lung epithelial cells led to an increase in the activity of the Krebs cycle and oxidative phosphorylation, stabilized the mitochondrial potential, conferred faster cell division, and lowered the levels of proapoptotic pseudokinase, TRIB3. Both oligos, mmu-CR805 and hsa-LDL1 conferred cross-species beneficial effects. Our data indicate a high degree of evolutionary conservation of retrograde signaling *via* a functional bit of the D-loop transcript, mito-ncR-805, in the mammals. This emphasizes the importance of the pathway and suggests a potential to develop this functional bit into a therapeutic agent that enhances mitochondrial bioenergetics.

## Introduction

Mitochondria are an integral part of eukaryotic cells. They have their own genome, which in mammals is approximately 16.6 kb, encoding 13 mitochondrial proteins of the electron transport chain (ETC) and harboring 22 tRNAs and 2 ribosomal subunit RNAs (Shadel and Clayton, 1997; Fernandez-Silva et al., 2003). The rest of the mitochondrial proteins, including proteins of tricarboxylic acid (TCA) or Krebs cycle) and ETC complexes, are encoded by nuclear genes, and are imported into the organelle (Scarpulla, 2008). Thus, signaling between the two organelles is necessary for mitochondrial integrity and cellular homeostasis. This signaling cascade is achieved through bidirectional communication between the nucleus and the mitochondria (Quiros et al., 2016; Vendramin et al., 2017). Nucleus-to-mitochondrion anterograde signaling is mediated by well-studied nuclear transcription factors, and many aspects of it are well characterized (Scarpulla, 2008). It is thought that anterograde signaling adjusts the functioning of healthy mitochondria to the cellular needs. When a cell faces stressful challenges, mitochondrial function may be compromised (Grek and Townsend, 2014; Zhang et al., 2019; Costiniti et al., 2020). Sometimes, the mitochondria can mitigate the damage caused by the stress, but sometimes that damage is irreversible. In either adaptive or maladaptive stress responses, the mitochondria need to convey their status to the nucleus, and often the composition and the function of the organelles need to be altered for cell survival (Scarpulla, 2008; Quiros et al., 2016; Vendramin et al., 2017). Retrograde mitochondrion-to-nucleus signaling is achieved through common metabolite intermediates, such as Ca^2+^ ions and TCA cycle metabolites (Quiros et al., 2016).

Nuclear genes that encode mitochondrial proteins (neMITO) monitor mitochondrial status. For example, the mitochondrial unfolded protein response (mito-UPR) is regulated by activating transcription factor associated with stress/activation transcription factor 5 (ATFS-1/ATF5). The ATFS-1 (or ATF5 in the mammals) is imported into the healthy mitochondria *via* its mitochondrion-targeting sequence (MTS). The ATFS-1/ATF5 that cannot be imported due to the effects of stress is transported into the nucleus to activate the genes to relieve that stress (Shpilka and Haynes, 2018; Munoz-Carvajal and Sanhueza, 2020). Another protein with mitochondrial localization, the DELE1, communicates with the mitochondrial stress to the integrated stress response *via* its relocation to cytosol (Fessler et al., 2020; Guo et al., 2020) to restore the mitochondrial function. Other neMITO proteins, such as the mitochondrial isomer of transcriptional coactivator G-protein pathway suppressor 2 (GPS2), act during physiological stresses (Cardamone et al., 2018; Kim K. H. et al., 2018). Nuclear translocation of GPS2 results in the synthesis of other neMITOs that change the composition and function of the mitochondria in a way that supports cell survival (Scarpulla, 2008).

The mitochondrial genome has recently shown to produce short mitochondrion-encoded peptides, such as MOTC-s and short humanin-like peptides (SHLPs), which are encoded in the 16S and 12S ribosomal RNA regions, respectively, of the mitochondrial genome (Yen et al., 2013; Cobb et al., 2016; Lee et al., 2016). The MOTC-s translocates into the nucleus to regulate the expression of the genes to optimize cellular metabolism, with implications in aging and muscle function (Reynolds et al., 2021). Other SHLPs may also act as important retrograde signaling molecules (Nashine et al., 2018; Nashine and Kenney, 2020).

In addition to protein factors, multiple non-coding RNAs have been shown to reside in mitochondria (Ro et al., 2013; Liang et al., 2021; Liu and Shan, 2021). These mito-ncRNAs are encoded either by the nuclear genome imported into the mitochondria or by the mitochondrial genome directly. An example of a nuclear genome-encoded mito-ncRNA is miR-146a-5p. In a brain injury model, the levels of miR146a-5p are decreased in the mitochondria and increased in the cytosol, where they regulate the uncontrolled activation of the NF-κB pathway (Wang et al., 2021). The mito-ncRNAs are not only encoded by the mitochondrial genome function within the mitochondria but also elsewhere, such as in the exosomes (Liang et al., 2021; Liu and Shan, 2021). Although their functions are not completely understood, they may regulate the maturation of mitochondrial transcripts (Ro et al., 2013). Circular mtDNA-encoded RNAs facilitate the import of nuclear-encoded proteins from both the sides of the organelle (Liu et al., 2020). The mtDNA-encoded long non-coding RNAs, sense non-coding mitochondrial RNA (SncmtRNA), and antisense non-coding mitochondrial RNA (ASncmtRNA), are generated from the transcripts derived from 12S and 16S mitochondrial rRNA genes. The SncmtRNA and ASncmtRNA exit into the cytosol, where they oppose each other to regulate the cell cycle progression and growth (Burzio et al., 2009; Borgna et al., 2017; Farfan et al., 2021). There is also evidence that mitochondrion-encoded tRNA-derived small fragments exit from the mitochondria under certain conditions, including cellular stress, and participate in different signaling pathways that operate between the mitochondria and the host cell (Liu et al., 2021; Meseguer, 2021). Two novel lncRNAs from the D-loop region were identified in the mammalian mitochondria: the H-strand transcript mitochondrial D-Loop (MDL1) and L-strand transcript MDL1AS. These were suggested to regulate some aspects of mtDNA replication but may also be processed into small RNAs with as-yet-unknown biological roles (Gao et al., 2018).

Therefore, accumulated evidence suggests that mitochondrion-localized factors, including mtDNA-encoded RNAs, are exported to convey mitochondrial status to the cell and the nucleus, leading to changes in the nuclear gene expression (Wen et al., 2019). We recently identified mtDNA-encoded small non-coding RNA, mmu-mito-ncR-805, which is generated from the D-loop of the L strand of the mitochondrial genome. The mmu-mito-ncR-805 is an abundant transcript that is stored in a granular form in the mitochondria under normal homeostasis and acts as a retrograde signal by translocating into the nucleus in certain cell types during the adaptive response to stress (Blumental-Perry et al., 2020). An increase in its nuclear localization correlates with an increase in the intermediates of the Krebs cycle and mitochondrial respiration and in the improved mitochondrial potential and function. It is to be noted that the SHLP2 and SHLP3 have biological effects similar to those of mmu-mito-ncR-805, suggesting that multiple retrograde signaling molecules, including mtDNA-encoded ncRNAs, modulate and fine-tune mitochondrial function under different physiological conditions and stresses (Nashine et al., 2018; Nashine and Kenney, 2020).

This work explores the feasibility of using mmu-mito-ncR-805 retrograde signaling pathways to enhance mitochondrial bioenergetics. To this end, short synthetic versions of an evolutionarily conserved region (CR) of mmu-mito-ncR-805, mmu-CR805, and a human ortholog of the CR, light-strand D-loop small non-coding transcript-1 (hsa-LDL1) (Ro et al., 2013) were tested for their ability to improve the mitochondrial energy production.

## Materials and Methods

### Cell Model

Immortalized mouse lung epithelial 12 (MLE12) and non-cancerous human bronchial epithelial immortalized with Ad12-SV40 2B (BEAS-2B) cell lines were purchased from the American Type Culture Collection and cultured as per their instructions (Wikenheiser et al., 1993) (US patent 4,885,238, dated December 05, 1989). The MLE12 cells were cultured in RPMI 1640 medium (Gibco, cat. #11875085) and BEAS-2B cells were cultured in DMEM/F-12 (Gibco, cat. #11320033), both supplemented with 4% of fetal bovine serum (Serum International, Laval, QC, Canada), insulin-transferrin-selenous acid (ITS) premix (BD Biosciences, San Jose, CA, United States), 10 nM of hydrocortisone (Sigma-Aldrich), 10 nM of estradiol (Sigma-Aldrich), 10 mM of 4-(2-hydroxyethyl)-1-piperazineethanesulfonic acid (HEPES), 2 mM of glutamine (CellGro), 100 U/ml of penicillin, and 100 g/ml of streptomycin. The BEAS-2B cells were grown using plates precoated overnight at 37°C with a mixture of 0.01 mg/ml of fibronectin (Sigma-Aldrich, cat. #F2006-5MG), 0.03 mg/ml of bovine collagen type I (Advanced Biomatrix, cat. #5005-100 ml), and 0.01 mg/ml of bovine serum albumin (heat shock fraction) with the pH of 5.2 (Sigma-Aldrich, cat. #A8022-100G) dissolved in Dulbecco’s Modified Eagle Medium (DMEM) (Corning, cat. #10-013-CM). All experiments were performed on cells between passages 2 and 12 (Kenche et al., 2013, 2016).

### Bioinformatics Analysis

We used the first 20 nt of mmu-mito-ncR-805 as a seed region (GAATTGATCAGGACATAGGG) in a BLAT search against the human nuclear and mitochondrial genomes (hg19 and NC_012920) and identified human mitochondrial homolog sequence, 16516-GAAGTAGGAACCAGATGTCG-16497. This human sequence was used to extend the species distribution analysis *via* the Multiz alignment algorithm available at the UCSC Genome Browser (Blanchette et al., 2004).

### Transfection Procedure

The MLE12 or BEAS-2B cells were seeded at the desired density (10,000 cells/well of 96-well plates, 40,000 cells/well of 24-well plates, and 150,000 cells/well of 6-well plates) in their respective media and left to adhere for 6–8 h. The medium was removed and replaced with OptiMEM. After 12 h, the cells were transfected with siPORT NeoFX (Life Technologies, Thermo Fisher Scientific) transfection reagent with 5 nM of RNA oligos (Table 1).

**TABLE 1:** List of oligos used in the study.

Oligo name	Oligo sequence (5′→3′), if available*11^a^*	Source, cat. #, and reference
Non-targeting RNA 1	Random sequence Pre-miR molecule extensively tested in human cell lines and tissues and validated to produce no identifiable effects on known miRNA function	Invitrogen, AM17110
Non-targeting RNA 2	GC medium-content oligos without homology to any known genes	Invitrogen, Universal control, 46-2001
Mmu-CR805	mG*mA*mA*UUGAUCAGGACAUAmG*mG*mG	Genscript
Mmu-ncR-805*^FL^*	mG*mA*mA*UUGAUCAGGACAUAGGGUUU- GAUAGUUAAUAUUAUAUGUCUUUCAAGUUC- UUAGUGUUUUUGGG*mG*mU*mU	Genscript, (Blumental-Perry et al., 2020)
Hsa-LDL1	GAAGUAGGAACCAGAUGUCG; mG*mA*mA*GUAGGAACCAGAUG*mU*mC*mG	Genscript
AI805	CCCUAUGUCCUGAUCAAUUC	Ambion, AM11866, (Blumental-Perry et al., 2020)
Mmu-CR805 FAM labelled	mG*mA*mA*UUGAUCAGGACAUAmG*mG*mG-6-FAM-3′	Genscript

^a^Some oligos were stabilized by modifications and additions to 5′ and 3′ termini, where “m” stands for 2-O-methyl-RNA and “*” stands for phosphorothioate.

Similar conditions were used for siRNAs targeting TRIB3, which was purchased from Ambion: Silencer Select predesigned siRNA of TRIB3 silencer (cat. #4390771, siRNA ID s105984 targeting RefSeq NM_175093.2 at 2 exons on location 256)^1^. A negative control oligo (Ambion, cat. #4390843) was used for siRNA control, as per the manufacturer’s instructions. After 6 h, the transfection complexes were removed, and the cells were incubated in their regular medium for further analysis.

### RNA Fluorescent *in situ* Hybridization

#### Probe Design and RNA-Fluorescent *in situ* Hybridization

To derive and detect mito-ncR-805, a 1ZZ probe (BA-Mm-mt-D-loop-O1-1zz) targeting 16,131–16,188 of NC_005089.1 was generated by ACD *via* ACD probe design software. The RNA-FISH was performed using the BaseScope Detection Red v2 assay (ACD, Inc.) according to the manufacturer’s protocol. The cells were seeded on coverslips (Fisherbrand, cat. #12-545-82 12CIR-1D) at a density of 40,000 cells/well, transfected or not with mmu-CR805, grown for 24 h, washed with phosphate-buffered saline (PBS), and fixed in 3.8% paraformaldehyde for 30 min at room temperature. Fixed cells were permeabilized, dehydrated in an ethanol series with PBS (50, 70, and 100% ethanol) for 1 min each at room temperature, and stored at −20°C until further use. The cells were treated with a 1:15 dilution of protease III provided with the kit for 7 min at room temperature. The cells were then hybridized with the probe, and amplification steps were performed according to the manufacturer’s instructions. The cells were then incubated with Hoechst solution (1 mg/ml in water; Sigma-Aldrich) to counterstain nuclei and mounted on a microscope slide. FastRed pigment was introduced into the amplification tree as a visual identifier of probe binding to enable both fluorescence and chromogenic detection.

#### Fluorescence Confocal Microscopy

For fluorescence detection, imaging was performed using a Leica SP8-HyVolution laser scanning confocal microscope (Leica Microsystems, Heidelberg, GmbH) equipped with an HCX PL APO 63×/1.4 oil immersion lens objective, HyD detectors, and Leica Application Suite X software. The mito-ncR-805-labeled and CR805-labeled structures were excited at 561 nm, with fluorescence emission collected from 570 to 630nm. Images were acquired at 1,256 pixels × 1,256 pixels and a step size of 0.15 μm. All images were acquired using identical microscope settings. Images were further processed using FIJI (ImageJ) for Mac version 2.1.0/1.53k (build 5f23140693) and Adobe Photoshop CS5.1 software.

#### Localization Analysis

To assess the localization of mmu-CR805 in different subcellular compartments, single planes were chosen for each image. The chosen plane contained the CR805 signals of the strongest intensities using the line scan function of FIJI (ImageJ). Line scans were obtained manually from multiple representative regions under each condition (specified in the figure legends). The Line scans were created using straight line and freehand line tools in FIJI software across the regions of interest: through the cytoplasm, through outlines of mito-ncR-805 areas, which were localized by proximity/partial colocalization with mitochondrial marker Tom20 (Blumental-Perry et al., 2020), and through the nucleus. The plot profile tool was used to determine the fluorescence intensities for the red channel along the line scan. Values were imported into Microsoft Excel, plotted as the function of distance in pixels (*x*-axis) and intensities (*y*-axis).

### Immunofluorescence Staining

The cells were seeded on coverslips at a density of 40,000 cells/well and transfected with FAM-labeled mmu-CR805 as described above. Twenty-four hours after transfection, the cells were fixed with 3.8% of paraformaldehyde for 20 min at room temperature, washed three times with PBS, and permeabilized with 0.05% of saponin-PBS. Non-specific staining was blocked by incubation with 5% of goat serum in 0.05% of saponin-PBS for 30 min at room temperature before incubating the cells for 2 h at room temperature with anti-Tom20 antibodies (Cell Signaling Technology, cat. #CS42406, at dilution 1:300). The cells were again washed three times, incubated with secondary antibodies conjugated to Alexa Fluor 568 (Invitrogen, cat. #A-11004, at dilution 1:500) for 1 h at room temperature, and washed twice with 0.05% of saponin-PBS. Nuclei were stained with Hoechst 33258 (Sigma-Aldrich, cat. #14530), and the cells were washed once with 0.05% of saponin-PBS and once with PBS and mounted on slides using Gold Antifade mounting medium (Thermo Fisher, cat. #P36930).

### Confocal Microscopy

Z-stack optical sections of 0.364 μm were acquired at a magnification of 100 times and zoom of 3.37 times using a Leica TCS SP8 (Leica Microsystems) equipped with an HC PL APO CS2 100×/1.4 oil immersion lens objective and hybrid detectors controlled by LAS X 3.5.7.23225 software. Tom20-labeled structures were excited at 578 nm, with an emission peak at 603 nm. FAM-labeled mmu-CR805 was excited at 495 nm, with an emission peak at 520 nm. Lightning Wizard was used for the acquisition of images, and for subsequent deconvolution processing of the images using Leica Lightning GPU-accelerated deconvolution. Green fluorescent carboxylate-modified microspheres, (505/515), 100 nm in diameter (Thermo Fisher, Invitrogen, cat. # F8888) were imaged at the identical conditions, and were used to calculate achieved resolution *via* full width at half maximum of the central peak of the intensity profile. The achieved resolution was calculated as 135 ± 16 nm. Individual experiments were performed with identical laser output levels, exposure times, and scaling.

### Line Scan Analysis

The line scan function of LAS X 3.5.7.23225 software was used to further determine the spatial proximity of green (FAM-labeled mmu-CR805) and red (Tom20) signals. Line scans were obtained manually from multiple representative regions under each condition (specified in the figure legends). The fluorescence intensities along the line scan for red and green channels and for green and blue (Hoechst) were imported into Microsoft Excel, plotted as a function of distance (nm) and intensities (Supplementary Table 1). Colocalization was confirmed when the intensity peaks of the green channel coincided with the intensity peaks of the red channel.

### RNA Extraction and Detection

RNA was extracted from MLE12 cells using an miRNeasy kit (Qiagen), which enriches the small non-coding RNAs, as per the manufacturer’s protocol. Northern blot analysis was performed using Signosis High Sensitive miRNA Northern blot assay kit (cat. #NB-1001 and 1002; Signosis, Inc., Santa Clara, CA, United States). Probes were 5′-biotin-CCCTATGTCCTGATCAATTC-3′ for the detection of mito-ncR-805 and 5′-biotin-ATCGTTCCAATTTTA GTATATGTGCTGCCGAAGCGAGCAC-3′for U6 (Signosis, cat. #MP-0512). Northern blots were probed with mito-ncR-805, stripped by incubating in 0.5% of sodium dodecyl sulfate (SDS) at 60°C for 60 min, and reprobed with U6 probe for a loading control.

### Real-Time RT-qPCR

Complementary DNA (cDNA) synthesis was performed using a high-capacity cDNA reverse transcription kit (Applied Biosystems, Foster City, CA, United States) on a T100 thermal cycler (Bio-Rad, cat. #186-1096). The following program was used for random primers cDNA synthesis: 25°C for 10 min, followed by 37°C for 2 h, and 85°C for 5 min. TaqMan^®^ miRNA Looped Primer cDNA synthesis was carried out using the same kit, using the following program: 16°C for 30 min, followed by 42°C for 30 min, and 85°C for 5 min. Primers were from TaqMan assays (Life Technologies and Thermo Fisher Scientific): for the transcripts containing CR of mito-ncR-805^1–20 nt^, cat. #4427975, the assay ID 002045 was used; for sno55 to normalize the expression levels of small RNAs, cat. #4427975, the assay ID 001228 was used; for TRIB3, Mm00454879-m1 was used; for RNR2, Mm04260181_s1 was used; and for GAPDH, Mm99999915_g1 (cytosolic marker and as a normalization of TRIB3 data) was used. All TaqMan assay qPCRs were performed using TaqMan Universal Master Mix II, no UNG, from Life Technologies (cat. #4440040) and a C1000 Touch Thermal Cycler chassis (Bio-Rad, cat. #1841100) equipped with a CFX96 real-time PCR detection system (Bio-Rad, cat. #1845097) and controlled by CFX Maestro software (Bio-Rad, cat. #12004110). The manufacturer’s standard program 10 min at 95°C, then 40 cycles for 15 s at 95°C, and 1 min at 60°C was used. Real time PCR results were compared using comparative *C*(*T*) methods and calculated as folds 2^–ΔΔ^*^C^*^t^ (Schmittgen and Livak, 2008). The relative abundance of mmu-CR805 in different fraction was calculated using identical inputs of RNA as Ct^fraction transfected^ − Ct^fraction total^ (Wang et al., 2020).

### Subcellular Fractionation

Separation of mitochondrial and cytosolic fractions was performed using a mitochondrial isolation kit from Sigma-Aldrich (cat. #MITOISO2) according to the manufacturer’s instructions for the isolation of the mitochondria from the cells with modifications. Briefly, the cells grown on plates were washed twice with PBS, scraped into lysis buffer, which is 1 time extraction buffer supplemented with protease inhibitory cocktail (Sigma-Aldrich Roche Biochemical Reagents) and 1:200 dilution of cell lysis solution, incubated 5 min on ice, and diluted with two volumes of 1 time extraction buffer. Lysates were centrifuged at 600 × *g* for 10 min at 4°C. One-third of the supernatant was removed for the analysis of the total fraction, which was further divided for protein analysis and RNA extraction. The remainder of the lysates were centrifuged at 11,000 × *g* for 10 min at 4°C. The supernatant was carefully removed and saved for further analysis as the cytosolic fraction, which was divided for protein and RNA analyses. Mitochondrial pellets were considered crude mitochondria and further purified by resuspension in 16% Percoll-PBS using the following Percoll gradient: 2 ml of 40% Percoll at the bottom, overlayed with 2 ml of 23% Percoll, followed by 16% of Percoll containing crude mitochondria. Percoll gradients were prepared using 1 time storage buffer provided in the mitochondrial isolation kit from Sigma-Aldrich as per the manufacturer’s instructions. The gradient was separated using SW41 Ti swinging bucket rotor 3,33,790 at 31,000 × *g* for 8 min at 4°C in a Beckman Coulter Optima XPN-100 ultracentrifuge.

The mitochondrial band was harvested at the lowest interface, diluted with four volumes of ice-cold 1 time storage buffer, and centrifuged in a fixed-angle Sorvall Legend RT centrifuge at 17,000 × *g* at 4°C for 10 min. The purified mitochondria were resuspended in 150 μl of 1 times storage buffer and treated with RNase I (Ambion, cat. #AM2294, 250 U/150 μl) at 4°C for 1 h to eliminate cytosolic RNAs. The treated mitochondria were suspended in Trizol for further RNA extraction and analysis.

### Nuclear Extracts and Nuclear RNA Preparation

The nuclear extracts were prepared by modification of the protocol by Wang et al. (2000). Briefly, MLE12 cells, collected by trypsinization, were resuspended in 10 mM of HEPES (pH 7.9), 10 mM of KCl, 1.5 mM of MgCl_2_, and 0.5 mM of dithiothreitol supplemented with protease inhibitory tablet (Sigma-Aldrich Roche Biochemical Reagents), incubated for 15 min on ice, followed by the addition of Non-idet P-40 to a final concentration of 0.325%. The cells were allowed to swell 10 min on ice with occasional shaking. Nuclear pellets were separated by centrifugation at 2,500 rpm for 4 min at 4°C. The nuclear pellets were washed once in the lysis buffer and resuspended in 20 mM of HEPES (pH 7.9), 0.45 M of NaCl, 1 mM of ethylenediaminetetraacetic acid (EDTA), 0.5 mM of dithiothreitol, 0.3 U/μl of RNase inhibitor (RNaseOUT recombinant ribonuclease inhibitor; Thermo, cat. #10777019), and 1 protease inhibitor tablet (cOmplete mini, EDTA free; Sigma-Aldrich Roche Biochemical Reagents) per 10 ml of lysis buffer. The nuclei were incubated with rocking at 4°C for 30 min. The nuclear extracts were cleared by centrifugation at 12,000 rpm for 10 min. The supernatant was divided for Western blot analysis and for RNA extraction and analysis. To eliminate cytosolic RNAs, the nuclear pellets were resuspended in the lysis buffer supplemented with 0.3 M of sorbitol and treated with RNase I (Ambion, cat. #AM2294, 100 U/100 μl) at 4°C for 1 h and suspended in Trizol for further RNA analysis.

### Protein Lysate Preparation and Western Blot Analysis

Western blotting was performed as described by Kenche et al. (2013). Briefly, the cells were either lysed in RIPA buffer (150 mM of NaCl, 1.0% of Non-idet P-40 or 0.1% of Triton X-100, 0.5% of sodium deoxycholate, 0.1% of SDS, 50 mM of Tris-HCl, pH 8.0) or the subcellular fractions were obtained as described above. Proteins were resolved by SDS-PAGE and transferred to nitrocellulose membranes (Bio-Rad, cat. #162-0115) by using a *Trans*-Blot Turbo transfer system (Bio-Rad, cat. #1704150) in *Trans*-Blot Turbo 5 times transfer buffer (Bio-Rad, cat. #10026938). High-molecular-weight proteins run using 1.5-mm of SDS gels were transferred using the setting of 25 V, 1.3 A constant, for 15 min (10 min for H3). Membranes were blocked in Tris-buffered saline (TBS) with 0.1% of Tween (TBST) supplemented with 5% of non-fat dry milk (Blotto; Santa Cruz Biotechnology, cat. #sc-2324) for 1 h at room temperature, washed with TBST twice, and incubated with primary antibodies in either TBST with 5% of bovine serum albumin or 5% of milk overnight at 4°C. The primary antibodies against the following were used: lactate dehydrogenase A (LDHA; Cell Signaling, cat. #2012, 1:1,000), succinate dehydrogenase subunit A (SDHA; Abcam, cat. #ab14715, 1:1,000), H3 (Cell Signaling, cat. #9715, 1:4,000), nucleophosmin (Abcam, cat. #ab10530 [FC82291], 1:1,000), β-actin (Abcam, cat. #ab6276, 1:10,000), TRIB3 (LSBio, LifeSpan BioSciences, cat. #LS-C164592, 1:250), and phospho-Akt (Trh308) (Cell Signaling, cat. #2965, 1:500). The blots were washed three times in TBST for 5 min each, incubated in TBST supplemented with 5% of milk with secondary antibodies for 1–2 h at room temperature, washed three times with TBST, and developed using Pierce ECL Western blotting substrate (Thermo Scientific, cat. #32106). The secondary antibodies included were goat anti-mouse IgG horseradish peroxidase (Thermo Fisher Scientific, cat. #31430, 1:5,000) and goat anti-rabbit IgG horseradish peroxidase (Thermo Fisher Scientific, cat. #31460, 1:5,000). Probed membranes were imaged using ChemiDoc MP imaging system (Bio-Rad, cat. #17001402) equipped with a blot/UV/stain-free sample tray for ChemiDoc MP/ChemiDoc imaging systems (Bio-Rad, cat. #12003028) and processed using Bio-Rad Image Lab version 6.1.0 (build 7 SE for Mac).

### Metabolic Labeling

The MLE12 cells were transfected with either CR805 or a non-targeting sequence; 30 h post-transfection, the cells were exposed to 20 mM [U-^13^C]glucose for 12 h and harvested. Metabolic flux was determined following ^13^C label incorporation into various metabolites. After incubation, 0.5 ml of removed medium was saved from one well of each condition and from each plate. The cells were washed while attached on the plates with PBS (three times, at room temperature wash, moving to 10°C, and completed with ice-cold PBS wash), fixed by adding 1 ml of 80% ethanol cooled at −20°C, collected by scraping and placing on dry ice ethanol bath, and stored at −80°C for further analysis.

### Assay of Medium [U-^13^C]Glucose Enrichment

Glucose isotopic enrichment was determined according to Gao et al. (2015) with modifications. Briefly, the glucose was extracted by the addition of 500 μl of ice-cold ethanol into 50 μl of a medium. Samples were mixed and incubated on ice for 30 min. The samples were centrifuged at 4°C for 10 min at 14,000 rpm, and ethanol was transferred to GC/MS vials and evaporated to dryness in a SpeedVac evaporator. Glucose was converted into its pentaacetate derivative by its reaction with 150 μl of acetic anhydride in pyridine (2:1 [vol/vol]) at 60°C for 30 min. The samples were evaporated to dryness, and the glucose derivative was reconstituted in 80 μl of ethyl acetate and transferred to a GC/MS insert. The samples were injected in duplicates, and the masses 331–337, containing M0–M5 isotopomers, were monitored. Enrichment was determined as a ratio of M5 to Σ_M0–M5_.

### Metabolite Extraction

The MLE12 cells were treated as previously described (Yang et al., 2008). Briefly, cell extract was vortexed and sonicated using an ultrasonicator alternating 30 s on 30 s off for 10 min. The cells were pelleted by centrifugation at 4°C for 10 min at 14,000 rpm. The supernatant was transferred to GC/MS vials and evaporated to dryness under a gentle stream of nitrogen. Keto and aldehyde groups were reduced by the addition of 10 μl of 1 N NaOH and 15 μl of NaB_2_H_4_ (prepared as 10 mg/ml in 50 mM of NaOH). After mixing, the samples were incubated at room temperature for 1 h, acidified by 55 μl of 1 N HCl (dropping the acid slowly), and evaporated to dryness. Fifty microliters of methanol were added to precipitate boric acid. Internal standard was added (10 μl of 17:0 formic acid, 0.1 mg/ml). The samples were evaporated to dryness and reacted with 40 μl of pyridine and 60 μl of *tert*-butylbis(dimethylsilyl) trifluoroacetamide with 10% trimethylchlorosilane (Regisil) *tert*-butyl(dimethylsilyl) at 60°C for 1 h. Resulting *tert*-butyl(dimethylsilyl) derivatives were injected into the GC/MS equipment.

### GC/MS Conditions

Analyses were carried out on an Agilent 5973 mass spectrometer equipped with a 6890 Gas Chromatograph. An HP-5MS capillary column (60 m × 0.25 mm × 0.25 μm) was used in all the assays, with a helium flow of 1 ml/min. The samples were analyzed in selected ion- monitoring mode using electron impact ionization. Ion dwell time was set to 10 ms. The following metabolites were detected: α-ketoglutarate, alanine, aspartate, citrate, fumarate, glutamate, lactate, malate, oxaloacetate, pyruvate, serine, and succinate.

### Calculations

Fractional metabolic flux was determined according to the relationship between the precursor ([U-^13^C]glucose) and the product ([^13^C]-labeled metabolites). Molar percent enrichment (MPE) of the metabolites was determined in the same manner as for glucose (refer to GC/MS conditions). Fractional metabolic flux was calculated as follows: MPE_product_/MPE_precursor_. The absolute metabolic rate shown in Figures 4A–C was determined as a product of the fractional metabolic flux and pool size, i.e., the relative concentration of the metabolite of interest (Supplementary Table 2).

### Mitochondrial Bioenergetics Analysis

The MLE12 cells were seeded at 10,000 cells/well (80 μl) and transfected with either CR805 or a negative control as described above. Forty hours after transfection and 1 h before the measurements, RPMI medium was changed to unbuffered DMEM (Seahorse Bioscience, United States) supplemented with 1% of fetal bovine serum, 4 mM of glutamine (Sigma-Aldrich), 2 mM of sodium pyruvate (Gibco), 10 mM of glucose (Sigma-Aldrich), 100 μM of insulin (Sigma-Aldrich), and mitochondrial bioenergetics were measured using a Seahorse XFe96 extracellular flux analyzer and the XF Cell Mito Stress kit (Seahorse Bioscience) as described before (Zhang et al., 2018). Real-time measurement of oxygen consumption rate was obtained as per the manufacturer’s instructions by sequential treatment with 1 μM of oligomycin, 1 μM of carbonyl cyanide *p*-trifluoromethoxyphenylhydrazone (FCCP), and 1 μM mixture of rotenone/antimycin A, and normalized to total protein amount measured by a Pierce BCA protein assay kit (Thermo Scientific). Basal respiration was calculated as the average of the first three readings before the addition of oligomycin minus the average of the last 3 readings after the addition of rotenone addition. Proton leak was calculated as the average from three readings after the addition of oligomycin minus the average of the last three readings. ATP production was calculated as basal respiration minus the proton leak. Maximal respiration was calculated as the average of the three readings after FCCP stimulation minus the average of the last three readings. Spare respiratory capacity was calculated as the maximal respiration minus the basal respiration. Coupling efficiency was calculated as 100× ATP production/non-mitochondrial respiration, where the non-mitochondrial respiration was the average of the last three readings after the addition of rotenone and antimycin A (Supplementary Table 3).

### Mitochondrial Membrane Potential Assays

Mitochondrial membrane potential was measured using a JC-10 mitochondrial membrane potential assay kit (Abcam, cat. #ab112134) according to the manufacturer’s instructions. The results were read by a BioTek Synergy HT spectrophotometer.

### Preparation of Cigarette Smoke Extract

The cigarette smoke extract (CSE) was prepared using the method described by Kenche et al. (2013). Research-grade 1R5F cigarettes (Kentucky Tobacco and Health Research Institute, Lexington, KY, United States) were used to prepare the CSE. The CSE was made fresh for each experiment by bubbling the smoke from 1 cigarette through 5 ml of serum-free medium in a 15-ml conical tube and filtering it through a 0.22-μm of filter to remove large particles and to maintain the sterility. This solution was designated 100% CSE and diluted in a culture medium to yield the concentrations specified for each experiment.

### Cigarette Smoke Extract Growth After Stress

Cells were plated at 50,000 cells/ml in 24-well plates. The cells were transfected as previously described; 20 h post-transfection, cells were exposed to 10% of CSE for 30 min. The medium was removed, the cells were washed twice with PBS, and fresh medium was added. The Cells were counted at time points specified in the figure legends.

### Statistical Analyses

Unless specified in text, data are expressed as means and standard deviations from at least three independent experiments. Fold-change for qPCR was determined using the 2^ΔΔ^*^CT^* method (Livak and Schmittgen, 2001; Schmittgen and Livak, 2008). Student’s *t*-tests were used to determine the *P*-values. *P*-values of ≤0.05 were considered statistically significant. ANOVAs were used to determine whether multiple groups differed from each other.

## Results

The first 20 nt of mmu-mito-ncR-805 are evolutionarily conserved, and the corresponding synthetic oligos are detected in multiple cellular compartments in the transfected cells. Our previous work demonstrated that the amount of mmu-mito-ncR-805 in the nucleus increased almost 10-fold in CSE-exposed cells. This increase coincided with the recovery from stress (Blumental-Perry et al., 2020). An antisense inhibitor of mito-ncR-805, AI805, revealed a positive regulation of a subset of neMITO genes and energy metabolism by mito-ncR-805 (Blumental-Perry et al., 2020). Accordingly, overexpression of a synthetic oligo corresponding to the full-length mmu-mito-ncR-805 (mmu-ncR-805^FL^) increased the expression of a few neMITO genes and had a beneficial impact on the intermediates of the Krebs cycle (Blumental-Perry et al., 2020). We therefore wanted to employ a forced overexpression system to test if we can improve the bioenergetics of the cells transfected with ncRNA.

**FIGURE 1:**
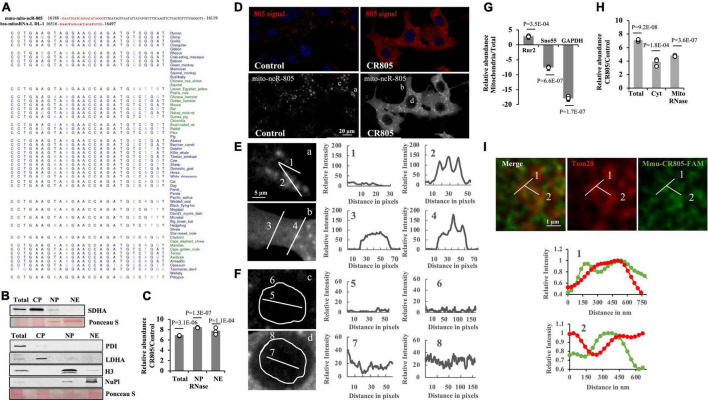
The first 20 nt of mito-ncR-805 is evolutionarily conserved in the mammals. **(A)** Alignment of mmu-mito-ncR-805 (accession number MI0005204) and hsa-mito-RNA-L-DL-1, which maps to the D-loop of the control region (Ro et al., 2013). Multiz alignment of hsa-mito-RNA-L-DL-1 with sequences from 100 vertebrates. The evolutionarily conserved region is shown in red. **(B–H)** Subcellular distribution of transfected mmu-CR805 (or control) in MLE12 cells. **(B,C)** Total, cytoplasmic (CP), nuclear pellet (NP), and nuclear extract (NE) fractions were prepared. NPs were treated with RNase I. **(B)** Purity of the fractions was assessed by the presence of the cytosolic protein, LDHA, mitochondrial protein SDHA, endoplasmic reticulum protein PDI, nuclear chromatin protein H3, and nucleoplasm protein nucleoplasmin *via* Western blot analysis. Ponceau S staining is shown as a loading control. **(C)** Small RNAs were extracted from generated fractions and analyzed for the expression levels of transcripts containing the conserved region of mito-ncR-805 by RT-qPCR. **(D)** MLE12 cells transfected with control or mmu-CR805 were grown on slides, fixed, and hybridized with a mito-ncR-805-specific probe. Images were acquired at 63 times magnification on a Leica DM IRE2 Leica SP8 laser confocal microscope. **(E,F)** Enlarged areas from Panel **(D)** are shown on the left. Graphs on the right show relative red channel fluorescence intensity through the line-scanned regions. **(E)** Line-scans drawn through cytoplasm (1 and 3) and structures positive for mmu-mito-ncR-805 and/or mmu-CR805 (2 and 4). **(F)** Line scans drawn through and around the perimeter of nuclei of cells transfected with non-targeting RNA (top image) and mmu-CR805 (bottom image). **(G,H)** MLE12 cells transfected with mmu-CR805 or non-targeting RNA oligos were lysed and analyzed (total fraction). Cytosolic fractions (Cyt) were obtained by separating the crude mitochondria, which were further purified using Percoll gradient and treated with RNase I to remove non-mitochondrial RNAs. The RNAs were isolated from the obtained fractions. **(G)** Purity of the fractions was evaluated by comparing the expression levels of mtDNA-encoded mRNA of Rnr2, nucleus-encoded and localized Sno55-RNA, and cytosolic GAPDH mRNA. Graph shows the difference between *C*_*T*_ levels relative to the total lysates and with identical RNA inputs. **(H)** Expression levels of transcripts containing the mmu-CR805 sequence in different fractions as evaluated by RT-qPCR. Graph shows the difference in *C*_*T*_ values between the control and mmu-CR805-transfected cells. **(I)** The MLE12 cells were transfected with FAM-labeled mmu-CR805, fixed, and stained for Tom20. Images were acquired at a magnification of 100 times and zoom of 3.37 times on a Leica TCS SP8 microscope with accelerated deconvolution (entire images are provided in Supplementary Figure 2). Line scans were drawn through Tom20-labeled structures. Graphs show relative red and green channel fluorescence intensity through the line-scanned regions.

The 70-nt mmu-mito-ncR-805 transcript is mouse-specific. To identify a possible human homolog, we analyzed the sequence and found that the first 20 nt were unique to the mitochondrial genome; the rest of the transcript shares short stretches of homology with two nuclear genes, ZFP280c and Adams9 (Blumental-Perry et al., 2020). We performed a BLAST analysis with the 20-nt region against the human mitochondrial genome and searched the existing databases for previously identified human transcripts that are generated from the D-loop of the L strand of the human mitochondrial DNA that shares a homology with the mito-ncR-805. The transcript, hsa-mitosRNA-L-DL-1 (Figure 1A) shares a high degree of homology with the first 20 nt of mito-ncR-805 (Ro et al., 2013). We therefore investigated whether there is any evolutionary conservation of this region in the mammalian mitochondrial genomes by using Multiz analysis, which demonstrated that the 20-nt sequence is conserved in the mammalian mitochondria (Figure 1A). Whether those homologous sequences are able to generate transcripts in other species is not known, but the conservation within the mitochondrial non-coding sequences is strongly suggestive of a functional importance of those transcripts. We therefore hypothesized that the first 20 nt of mmu-mito-ncR-805 may be critical for its function. The first 20 nt of mmu-mito-ncR-805 was introduced into the cells in the form of the single-stranded RNA oligo, mmu-conserved region 805 (mmu-CR805), titrated to transfect at least 80% of the cells (5 nM). Northern blot analysis confirmed the presence of the 20 nt of mmu-mito-ncR-805 in the transfected cells (Supplementary Figure 1A).

We next wanted to observe the intracellular distribution of the transfected oligo. Since our previous data suggested that mmu-mito-ncR-805 has a nuclear function, we first evaluated if the mmu-CR805 oligo is found in the nuclei of the transfected cells. Figure 1B demonstrates that the isolated nuclear pellets treated with RNase had small amounts of detectable SDHA, as expected, whereas the nuclear extracts did not. Both the RNase-treated nuclear pellets and the extracts from the transfected cells demonstrated a significant increase in mmu-CR805 signal (Figure 1C). Since fractionation involves enrichment, we verified these findings using a microscopy in two different ways. First, we used FISH and demonstrated an increase in the signal following the forced expression of mmu-CR805 (Figure 1D). Line scan analysis showed increasing red signal intensity (from 10–25 relative units in the background to 45–85 in the transfected cells), mostly in the cytosol of the transfected cells (Figure 1E, 1 and 3, and Supplementary Table 1). Our previous work demonstrated that the endogenous mmu-mito-ncR-805 localized into the structures that partially overlap with mitochondrial marker Tom20 and hence their presence requires intact mitochondria (Blumental-Perry et al., 2020). The signal intensity of those structures increased only slightly in the transfected cells (compare Figure 1E, 3 and 4). This may be due to the signal saturation and it was addressed in experiments presented below. Figure 1F demonstrates that endogenous levels of mmu-mito-ncR-805 in the nucleus are below FISH detection in the cells transfected with a non-targeting oligo (Figure 1F, c, 5 and 6), whereas mmu-CR805 was detectable (Figure 1F, d, 7 and 8) and its signal increased from 15 (background) to 45–50 units, suggesting that the mmu-CR805 oligo entered the nucleus. Similar results were obtained using fluorescently labeled mmu-CR805 oligos (Supplementary Figures 2D,E). Therefore, these data provide strong evidence in support of the nuclear localization of the transfected mmu-CR-805.

To further assess the ability of mmu-CR805 to enter into different cellular compartments, we isolated cytosolic and enriched (purified on Percoll gradient and RNase treated) mitochondrial fractions (refer to Figure 1G and Supplementary Figure 1B for the fraction purity). The mitochondrial fraction demonstrated very low RT-qPCR signal for the nuclear marker, sno55, whereas there was a significant increase in the mmu-CR805 signal (Figure 1H). We next used accelerated deconvolution confocal microscopy to gain sufficient resolution of 120-150 nm to determine if Tom20 and FAM-labeled mmu-CR805 oligo signals overlap. Imaging demonstrates multiple yellow dots, and Line Scan analysis demonstrates a complete overlap between Tom20 and CR805 for the stretches of at least 250–300 nm at multiple sites (Figure 1I and Supplementary Figure 2). Our data suggest that some mmu-CR805 oligos were localized into the mitochondrial structures labeled by Tom20. Nevertheless, the forced expression of RNA oligos, such as mmu-CR805 represent a good system to study the consequences of non-mitochondrial evolutionarily conserved functional bits of mmu-mito-ncR-805.

### RNA Oligos Containing Conserved Region of Mito-ncR-805 Are Biologically Active

**FIGURE 2:**
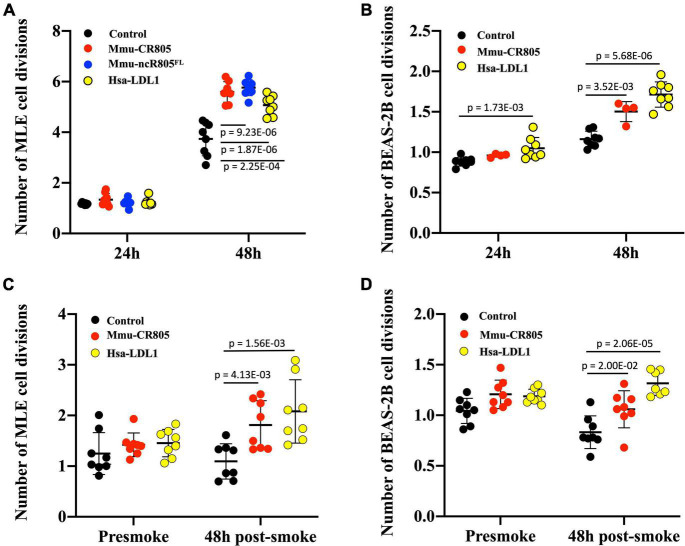
RNA oligos containing the conserved regions of mouse and human orthologs (mmu-CR805 and hsa-LDL1, respectively) demonstrate biological activity similar to that of the full-length ncR-805 in MLE12 and BEAS-2B cells. The MLE12 **(A,C)** and BEAS-2B **(B,D)** cells were transfected with non-targeting RNA, mmu-CR805, or hsa-LDL1. The MLE12 cells were also transfected with mmu-ncR-805^FL^. **(A,B)** Growth rates of cells 24 and 48 h post-transfection. **(C,D)** Growth rates of cells post-stress. Twenty-four hours post-transfection, the cell numbers were counted and the cells were exposed to 10% of CSE for 30 min, washed, and allowed to recover in their respected fresh media. Forty-eight hours post-exposure, the cell numbers were counted again.

Since we focused on the delivery of a “functional bit” of mmu-mito-ncR-805 and its human ortholog, we wanted to see if those oligos have any biological activity in the transfected cells. According to our previous research (Blumental-Perry et al., 2020), the mmu-mito-ncR-805 accelerates the repopulation of cells lost after the stress from smoke, presumably due to the higher bioenergetics of cells with high levels of mmu-mito-ncR-805 and their ability to divide faster (Blumental-Perry et al., 2020). We therefore introduced mmu-CR805, its human ortholog hsa-LDL1, and the full-length ncR-805^FL^ into MLE12 cells and mmu-CR805 and hsa-LDL1 into BEAS-2B cells and compared their growth rates with and without stress. Figures 2A,B demonstrate that over 48 h, all the mmu-mito-ncR-805 derivatives and the human orthologs induced faster cell division of MLE12 and BEAS-2B cells, with respective orthologs being slightly less efficient than species-specific oligos but still significantly different from cells transfected with non-targeting oligos. Importantly, the growth rates of MLE12 cells carrying the functional bit oligo, mmu-CR805, and the full-length synthetic transcript, ncR-805^FL^, were the same, supporting the idea that the 20-nt conserved region of mmu-mito-ncR-805 is indeed a functional bit. Next, the MLE12 and BEAS-2B cells were stressed by short-term exposure to CSE (30 min, 10% of CSE). This exposure was titrated to cause the death of approximately 50% of the cell population. Both the mouse and human orthologs conferred faster cell division following stress (Figures 2C,D). Therefore, we concluded that the first 20-nt segments of mmu-mito-ncR-805 and its human orthologous transcript are biologically active and function similarly to mito-ncR-805, which justified the functional studies that follow.

### CR805 Increases Tricarboxylic Acid Cycle Intermediates

**FIGURE 3:**
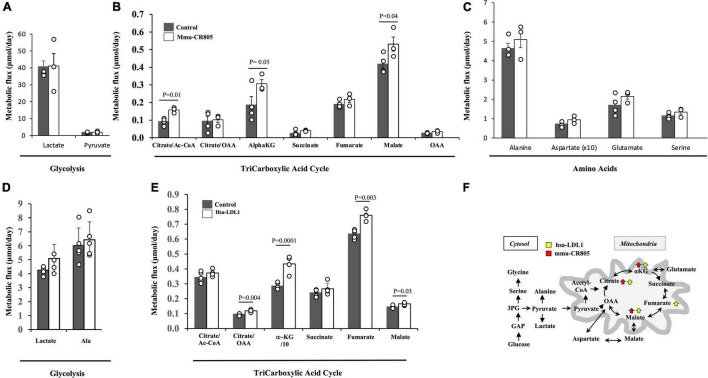
mmu-CR805 and hsa-LDL1 increase the TCA cycle activity. **(A–C)** The MLE12 cells were transfected with either 12 nM mmu-CR805 or a non-targeting sequence. **(D,E)** The BEAS-2B cells were transfected with either hsa-LDL1 or a non-targeting sequence. At 30 h post-transfection, the cells were exposed to 20 mM of [U-^13^C]glucose for 12 h and harvested, and the metabolic flux was determined according to ^13^C label incorporation into various metabolites. Metabolic flux of glycolysis **(A,D)**, TCA cycle activity **(B,E)**, and amino acid turnover **(C)**. Metabolic flux was determined as a product of fractional flux multiplied by the concentration (pool size) of a metabolite. Fractional flux was calculated as ^13^C enrichment of a product metabolite over the [^13^C] enrichment of the precursor, i.e., [U-^13^C]glucose. All quantifications are presented as means from at least four independent experiments ± S.E.M (Supplementary Table 2). Graphs are representative of at least two independent biological experiments of four replicates. **(F)** Schematic representation of the major findings on the glucose metabolic flux regulated by the forced overexpression of mmu-CR805 and hsa-LDL1.

We previously demonstrated that mmu-mito-ncR-805 can be found in the nuclei of cells recovering from stress and is associated with an increase in the mRNA levels of genes for multiple subunits of ETC complexes and metabolic enzymes, including the enzymes of the TCA cycle and related pathways (Blumental-Perry et al., 2020). We therefore examined the metabolic flux of the TCA intermediates in MLE12 and BEAS-2B cells transfected with mmu-CR805 and hsa-LDL1, respectively, by direct measurements of metabolic flux using stable isotope incorporation and mass isotopomer analyses. Neither mmu-CR805 nor hsa-LDL1 affected the glycolytic flux, as seen from the small changes in alanine and lactate (Figures 3A,C,D); however, they increased the absolute flux of TCA intermediates (Figures 3B,E, and Supplementary Table 2). Moreover, the significant increase in fumarate/malate is indicative of further channeling of energy to increase pyruvate production *via* the so-called mitochondrial gas pedal (Gellerich et al., 2013). This corresponds to the same response we previously characterized in the MLE12 cells recovered from CSE exposure (Blumental-Perry et al., 2020). Therefore, the transfection with 20 nucleotides of mito-ncRNA-805 is sufficient to promote a metabolic response (Figure 3F).

**FIGURE 4:**
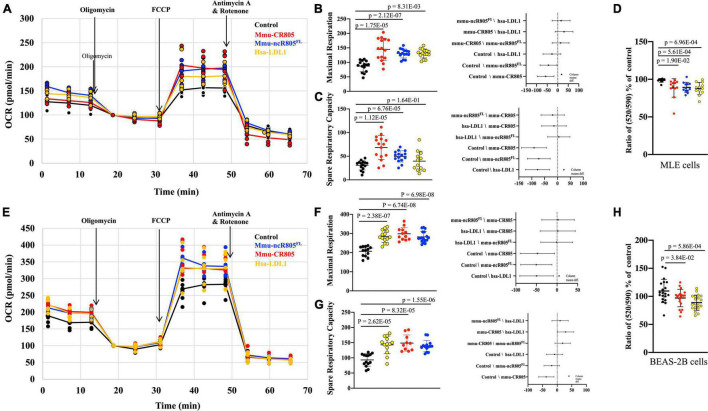
mmu-CR805 and hsa-LDL1 increase mitochondrial bioenergetics. The MLE12 **(A–D)** and BEAS-2B **(E–H)** cells were transfected with indicated CR805-containing RNA oligos or non-targeting RNA; at 48 h post-transfection, bioenergetics were measured using a Seahorse XFe96 extracellular flux analyzer, and mitochondrial membrane potential was measured using JC-10 assay. Oxygen consumption rate (OCR) was measured at the basal level and with subsequent and sequential additions of oligomycin (1 μM), FCCP (1 μM), and rotenone (1 μM) + antimycin A (1 μM) in MLE12 **(A)** and BEAS-2B **(E)** cells. Graphs are representative of experiments with at least three biological repetitions (Supplementary Table 3). Graphs of maximal mitochondrial respiration in MLE12 **(B)** and BEAS-2B **(F)** cells and spare respiratory capacity for MLE12 **(C)** and BEAS-2B **(G)** cells. Results from ANOVAs of differences between the groups are shown. **(D,H)** Mitochondrial membrane potential determined by a JC-10 assay, where monomer/aggregate ratios specify the depolarization of the mitochondrial membrane. All constructs containing CR805 or its human ortholog region had a lower monomer/aggregate ratio and increased ΔΨm.

### CR805 Increases Mitochondrial Bioenergetics Activity

We next used the XF Cell Mito Stress kit to measure the mitochondrial oxygen consumption rate in MLE12 cells at baseline and in response to the overexpression of different mmu-mito-ncR-805 oligos or their human orthologs of the functional bits (Figure 4A). We found that all tested oligos increased the maximal mitochondrial respiration and spared the respiratory capacity (Figures 4B,C and Supplementary Table 3). The rates of non-mitochondrial respiration were not affected, which was in agreement with the metabolomic data from the previous section, and demonstrated that it is not influenced by mmu-CR805 or by hsa-LDL1 (Figures 3, 4A).

High activity of the enzymes of the TCA cycle, reflected in our metabolomic data, and ETC, as indicated by the Mito Stress test results, should result in a higher mitochondrial membrane potential (ΔΨm). We therefore measured ΔΨm using a JC-10 assay (Reers et al., 1991) and found that the MLE12 cells expressing mmu-CR805 or hsa-LDL1 had lower depolarization of the mitochondrial membrane (higher ΔΨm) (Figure 4D). A similar analysis was performed in BEAS-2B cells either transfected with or not with mmu-ncR-805^FL^, mmu-CR805, or hsa-LDL1. The mmu-ncR-805^FL^, mmu-CR805, and hsa-LDL1 increased the maximal mitochondrial respiration, spared the respiratory capacity and the mitochondrial membrane potential (Figures 4E–H). In conclusion, both the mitochondrial respiration and the membrane polarization are increased by forced overexpression of the CR of mmu-mito-ncR-805 or its human ortholog in both the murine and human alveolar epithelial cells.

### High Levels of mmu-CR805 Result in Low mRNA and Protein Levels of Pro-apoptotic Pseudokinase TRIB3

**FIGURE 5:**
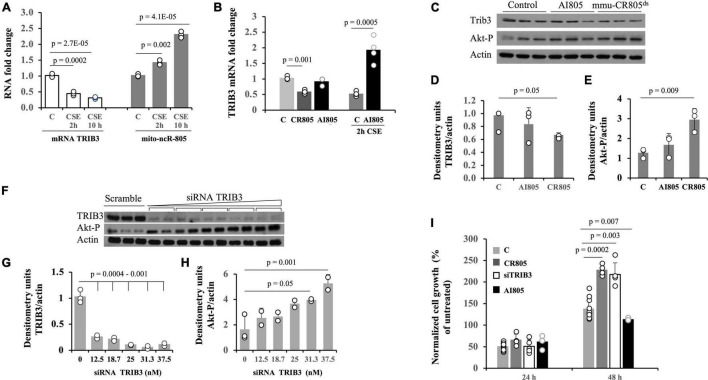
Forced expression of mmu-CR805 results in lower abundance of Akt kinase inhibitor, TRIB3 mRNA. **(A)** The MLE12 cells were either exposed or not exposed to CSE, the RNA was extracted, and the expressions of TRIB3 and mmu-mito-ncR-805 were tested by RT-qPCR. **(B–E)** The MLE12 cells were transfected with mmu-CR805, AI805, or non-targeting RNA and were either stressed or not on exposure to CSE, and RNA or proteins were extracted to assess the expression of TRIB3. **(B)** Levels of TRIB3 mRNA as evaluated by RT-qPCR. **(C)** Levels of TRIB3 protein and Akt1 phosphorylation as evaluated by Western blotting. Graphs represent quantification of TRIB3 protein levels **(D)** and Akt1 phosphorylation **(E)**. **(F–H)** The MLE12 cells were transfected with either increasing concentrations of siRNA against TRIB3 (in duplicates [TRIB3 Silencer Select S105984]) or with non-targeting siRNA, and the efficiency of TRIB3 depletion was tested by evaluating TRIB3 protein levels *via* Western blotting. Since TRIB3 inhibits the activation of prosurvival kinase Akt1, the Akt kinase activation/phosphorylation was used as an additional parameter to evaluate TRIB3 downregulation. Actin was used as a loading control. Graphs represent quantification of TRIB3 protein levels **(G)** and Akt1 phosphorylation **(H)**; 25 μM of siRNA against TRIB3 was chosen for further experiments. **(I)** The MLE12 cells were transfected with mmu-CR805, siRNA-TRIB3, AI805, or non-targeting RNA; 24 h post-transfection, the cell numbers were counted to ensure that pre-smoke cell numbers did not differ between the groups. No significant changes were observed, as expected from the data presented in Figure 2A. Twenty-four hours post-transfection, the cells were either exposed or not to 10% of CSE for 30 min, washed, and returned to grow in their regular medium; the cell counts were recorded 24 and 48 h after exposure to CSE. All experiments are representative of three independent experiments performed with triplicate samples.

Akt1 kinase is activated during the stress of smoking (Nakayama et al., 2002; Nicoletti-Carvalho et al., 2010). The Akt1 stress-related activation is not a common feature of stress response, where downregulation of prosurvival Akt1 activity is expected, but a response is specific to the stress from smoke and nicotine exposure. It can have numerous effects, including but not limited to increased mitochondrial bioenergetics (Li et al., 2013). We previously found that a decrease in the mRNA levels of the Akt1 inhibitor pseudokinase, TRIB3 (Nicoletti-Carvalho et al., 2010; Mondal et al., 2016) coincides with the recovery of cells from the stress due to smoke and with an increase in the levels of mmu-mito-ncR-805 in MLE12 cells (Figure 5A) (Blumental-Perry et al., 2020). We therefore asked if the bioenergetic effect of mmu-CR805 may culminate in the lower levels of TRIB3 mRNA. Forced expression of mmu-CR805 indeed resulted in a decrease in the levels of TRIB3 mRNA and protein as well as an increase in Akt1 phosphorylation (Figures 5B–E). Depletion of mmu-mito-ncRNA-805 with antisense inhibitor, AI805 [refer Supplementary Figure 3 and (Blumental-Perry et al., 2020)] caused an increase in the expression of TRIB3 mRNA, but only in the stressed cells (Figure 5B). Without stress, the cells were able to function with lowered levels of mmu-ncR-805 without any correlation with TRIB3 levels (Figures 5C,D and Supplementary Figure 3).

We compared the recovery after acute CSE exposure of MLE12 cells transfected with non-targeting RNA, mmu-CR805, siRNA-TRIB3 (Figures 5C–H), and AI805. Both mmu-CR805 and siRNA-TRIB3 promoted post-stress cell growth, with mmu-CR805 being more efficient (Figure 5I), supporting the idea that mmu-CR805 has broader downregulation than that of the effects of TRIB3 mRNA. As expected, cells carrying AI805 grew slower after stress (Figure 5I). Therefore, one of the actions of mito-ncR-805 may culminate in the downregulation of TRIB3 expression, which in turn promotes higher activity of the Akt1 (Mondal et al., 2016) and cell health.

## Discussion

Mitochondrial stress and malfunction are common features of multiple human diseases, including cardiovascular, pulmonary, and neurodegenerative diseases (Mizumura et al., 2014; Galluzzi et al., 2016; Annesley and Fisher, 2019; Bader and Winklhofer, 2020; Jusic et al., 2020). Here, we demonstrated that the forced expression of the evolutionarily conserved fragments of mtDNA-encoded mmu-mito-ncR-805, mmu-CR805, and its human ortholog, hsa-LDL1, improves the mitochondrial metabolism and bioenergetics. Our findings suggest that mitochondrial ncRNAs, which were proposed to act as a communicator between different cellular compartments, can be considered as a potential therapeutic target in the restoration of mitochondrial function (Liang et al., 2021).

Many efforts were and are directed toward improving the mitochondrial function in multiple diseased states and during aging (Corona and Duchen, 2016; Brown et al., 2017; Tampaki et al., 2018; Zhang et al., 2020). These efforts can be summarized in a simplified way as the ones that improve mitochondrial function, such as small molecules and ligands that can activate transcription factors for anterograde signaling, such as NRF1 and PPARα, Ca^2+^ ion modulators, and cardiolipin targeting and protecting compounds (inner mitochondrial membrane-specific lipid cardiolipin is needed for the correct assembly and function of ETC complexes). Some of them are reported to be safe, but their efficacy needs further investigation and improvement. The other class of mitochondrion-targeting compounds includes ones that inhibit mitophagy, mito-fission, and the mitochondrial permeability transition pore (Brown et al., 2017). Attempts to use those to prevent mitochondrial loss produced controversial results, probably due to the physiological corrections of malfunctioning mitochondria, and more research is needed to assess their potential use.

Retrograde signaling molecules that are generated in and released from the mitochondria during mitochondrial stress can lead to adjustment and, at proper circumstances, lead to a successful restoration of mitochondrial function. Recent discoveries support this notion (Landerer et al., 2011; Kim K. H. et al., 2018; Kim S. J. et al., 2018; Blumental-Perry et al., 2020). Those molecules input the functional state of mitochondria into the cellular homeostasis network by activating the mechanisms outside the mitochondria. It is to be noted that the open reading frames of such molecules, including mitochondrial ORF of the 12S rRNA type-c (MOTS-c) peptide, as well as SHLPs, are embedded within the mitochondrial RNR genes, the expression of which is tightly regulated and influences many aspects of the transcription and translation of the mitochondrial genome. Those peptides are currently in use as food supplements to prevent mitochondrial decline (Reynolds et al., 2021). The mito-ncR-805 is generated from the control region of the D-loop of the L strand of mtDNA, and SncmtRNAs and ASncmtRNAs are the recombined products of RNR genes (Landerer et al., 2011). Few up-to-date discovered retrograde signaling molecules are generated from the regulatory regions of the mitochondrial genome, strongly implying their governing function and importance in the communication between the genomes.

Small regulatory ncRNAs encoded by the mitochondrial genome (Rackham et al., 2011; Vendramin et al., 2017; Larriba et al., 2018) were suggested to be involved in communication between the mitochondria and the nucleus (Liang et al., 2021). For example, the mito-lncRNA, SncmtRNA, is observed in both the mitochondria and the nucleus and is shown to function in retrograde signaling (Landerer et al., 2011). We recently identified mito-ncR-805, which is generated from the light-strand promoter (LSP) in the D-loop regulatory region of mtDNA and relocates to the nucleus during adaptive stress. Conservation of non-coding sequences of the mitochondrial genome is low (Jansen, 2000; Zardoya, 2020). Accordingly, we found that an entire 70-nt mmu-mito-ncR-805 transcript is mouse-specific, but its first 20 nt demonstrates evolutionary conservation in the mitochondria of mammals. We therefore suggest that the 20 nt represents a functional bit, whereas the rest of the sequence has other functions, which are not conserved at the level of the sequence. To initiate studies into the possible function of the conserved region of mito-ncR-805, we overexpressed the CR of the mouse sequence as well as its human ortholog in both the mouse and the human cells, and evaluated the activity of the Krebs cycle, mitochondrial respiration, and the mitochondrial membrane potential. The forced expression of either mmu-CR805 or hsa-LDL1 increased those parameters in both the mouse and the human cells, although the ortholog oligos were somewhat less efficient than the species-specific ones. It is to be noted that when forcedly overexpressed, the magnitude of the effect of the full-length mouse transcript was very similar to that of the biological effects of its functional bit, mmu-CR805. On the basis of this observation, we concluded that the rest of the transcript may have either regulatory or stabilizing functions. Future research is needed to interrogate this hypothesis. Our findings support the idea that this region has functional significance and provide justification for future studies on the precise mechanism of the action of those functional bits in the mouse and human cells.

The forced overexpression system used in the present study did not enable us to determine the cellular compartment where the transfected oligo was active, because we detected it in the cytosol, the nucleus, and in some mitochondria. However, our previous data provided strong supporting evidence that mito-ncR-805 acts as a retrograde signaling molecule during stress from smoke, and its nuclear presence leads to changes in the expression of nuclear genes that encode mitochondrial proteins (Blumental-Perry et al., 2020). Indeed, the transcript levels of at least 14 nuclear genes that encode mitochondrial proteins were affected by the levels of mmu-mito-ncR-805. Those genes mostly encode the subunits of ETC complexes. Their induction by mmu-mito-ncR-805 and the subsequent increase in proteins they encode in the mitochondria can potentially explain the increase in the mitochondrial bioenergetics observed as a biological consequence of forced expression of functional bits of mmu-mito-ncR-805 or of its human ortholog, hsa-LDL1. Alternatively, there is a possibility that the transfected oligos are active in some mitochondrial compartment. We have not observed significant effects of the inhibition of mmu-mito-ncR-805 by AI805 on mtDNA replication or steady-state mitochondrial transcription or translation (Blumental-Perry et al., 2020). Nevertheless, the mmu-CR805 or its ortholog can potentially influence the activity of the ETC or affect the rates of transcription. Future research is needed to address this possibility.

The molecular mechanisms of CR805 function are unknown and need further investigation. Exposure to cigarette smoke is known to increase Akt1 activation (Nakayama et al., 2002). We associated the high levels of mito-ncR-805 with low expression of the pseudokinase TRIB3. We therefore, used TRIB3 levels as read out of CR805 activity. The TRIB3 is a multifunctional scaffolding protein that has been shown to coordinate multiple cellular signaling systems in a content-specific manner that can determine cellular fate (Ord and Ord, 2017; Stefanovska et al., 2021). We tested if a possible cellular outcome of high mmu-ncR-805 and CR805 is a low abundance of TRIB3 mRNA and protein. Indeed, high levels of CR805 resulted in lower levels of TRIB3. Cells with forced expression of CR805 had higher maximal respiration and spare respiratory capacity and grew faster than the control cells. Therefore, low TRIB3 mRNA is likely a result of a favorable cellular bioenergetic state induced by high levels of mmu-CR805. Cells with lowered levels of mito-ncR-805 (*via* the AI805 antisense inhibitor) had the same TRIB3 levels under unstressed conditions as cells with normal levels of mito-ncR-805. But low expression of mito-ncR-805 results in low maximal and spare respiratory capacities (Blumental-Perry et al., 2020), indicative of cells being close to their bioenergetics limit (Wang et al., 2018). When such cells experience stress, the levels of TRIB3 are significantly induced. Therefore, the action of mito-ncR-805 via its CR likely culminates in the coordination with other cellular systems important for mitochondrial function.

Interestingly, one of the TRIB3 functions is to inhibit Akt activation (Nicoletti-Carvalho et al., 2010). Akt1, when phosphorylated and activated in some systems, translocates into mitochondria and localizes within their membranes, where it can phosphorylate a number of mitochondrial residence proteins, including the α and β subunits of ATP synthase (Li et al., 2013). It would be interesting to see if mmu-CR805 contributes to increased mitochondrial respiration by reducing TRIB3 mRNA and thus enabling Akt1 to increase the activity of ETC complexes.

In summary, it is tempting to suggest that mito-ncR-805 serves as a mitochondrion-derived signaling molecule that has evolved to trigger adaptive cellular responses through increased bioenergetics. We speculate that this adaptive pro-energy molecule might be used to mitigate mitochondrial malfunction common to multiple human diseases.

## Data Availability Statement

The original contributions presented in the study are included in the article/Supplementary Material, further inquiries can be directed to the corresponding author/s.

## Author Contributions

TM participated in the design of the experiments, performed fractionation, western blot analysis, transfections, cell counting, some seahorse experiments, participated in analysis of the images, and in the preparation of the manuscript. DT, Z-WY, and JZ conceived and analyzed bioenergetic experiments. VL performed evolutional conservation analysis. WS designed, performed, and participated in the analysis of high-resolution imaging. IB and EP performed metabolomics analysis. RJ completed Northern blot analysis and participated in the interpretation of the results. JF performed RT-qPCR analysis. MD’A performed the titration of the transfection efficiency. MH and YP participated in the project design and interpreted multiple results and participated in writing and editing of the manuscript. AB-P conceived, designed, and coordinated the research project, performed multiple experiments, generated and interpreted the data, supervised all aspects of the study and the manuscript. All authors contributed to the article and approved the submitted version.

## Conflict of Interest

The authors declare that the research was conducted in the absence of any commercial or financial relationships that could be construed as a potential conflict of interest.

## Publisher’s Note

All claims expressed in this article are solely those of the authors and do not necessarily represent those of their affiliated organizations, or those of the publisher, the editors and the reviewers. Any product that may be evaluated in this article, or claim that may be made by its manufacturer, is not guaranteed or endorsed by the publisher.

## Footnotes

1
https://www.thermofisher.com/order/genome-database/details/sirna/s105984?CID=&ICID=&subtype=#assay-details-section

## Supplementary Material

Supplementary Figure 1 **(A)** The RNA enriched with small RNAs was isolated, resolved on 15% of urea gels, and hybridized with probes complementary to the conserved region of mmu-mito-ncR-805. The MTHe membrane was stripped and reprobed with a U6-specific probe as a loading control. **(B)** The MLE12 cells were transfected with mmu-CR805 or non-targeting RNA and lysed, and the lysates were analyzed (total). Cytosolic fractions were obtained by separating crude mitochondria, which were further purified using Percoll gradient and treated with RNase I to remove non-mitochondrial RNAs. The RNAs were isolated from obtained fractions. Purity of the fractions was evaluated by comparing the relative expression levels of mtDNA-encoded mRNA of Rnr2, nucleus-encoded and localized Sno55-RNA, and cytosolic GAPDH mRNA with identical RNA inputs.

Supplementary Figure 2 The MLE12 cells were transfected with FAM-labeled mmu-CR805, fixed, and stained for Tom20. Images were acquired at a magnification of 100 times and zoom of 3.37 times on a Leica TCS SP8 microscope with accelerated deconvolution. **(A)** Single plane of representative cell images, with two boxed areas that contain Tom20-labeled structures (1–2 and 3) and one boxed area through the nucleus (4). **(B)** Enlarged area 3 from **(A)**. **(C)** Graphs of relative red and green channel fluorescence intensity through the line-scanned region shown in **(B)**. **(D)** Enlarged area 4. **(E)** Graphs of a relative blue and green channel fluorescence intensity through the line-scanned region shown in **(D)**. **(F)** Line scans 5a–c were drawn to obtain the relative green channel intensity (FAM-labeled mmu-CR805) through cytoplasmic regions.

Supplementary Figure 3 Efficiency of inhibition by AI805. The MLE12 cells were transfected with AI805 or non-targeting RNA; 24 h post-transfection, the RNA was extracted and analyzed for the expression levels of mmu-CR805 containing transcripts.

Supplementary Table 1 Linescan analysis of green FAM-labelled mmu-CR805 and red (Tom20) signals.

Supplementary Table 2 Metabolic analysis of TCA intermediates.

Supplementary Table 3 Mitochondrial stress test kit data analysis.
