# Supplementary figures and images for: A Synthetic Small RNA Homologous to the D-Loop Transcript of mtDNA Enhances Mitochondrial Bioenergetics
Source: Front Physiol. 2022 Apr 6;13:772313. doi: 10.3389/fphys.2022.772313 (PMC9020786; doi:10.3389/fphys.2022.772313)

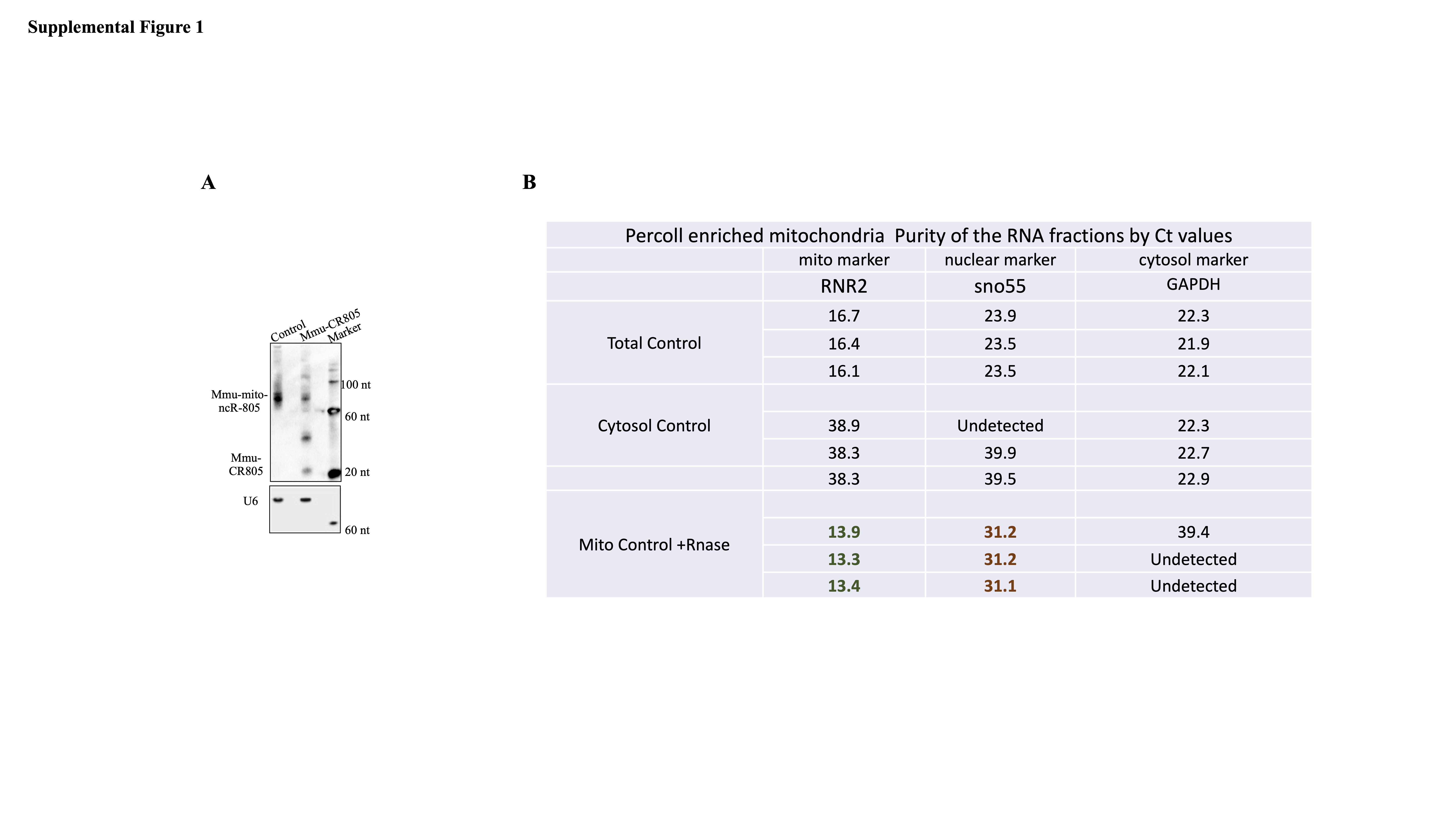

Supplement: Supplementary Figure 1 — (A) The RNA enriched with small RNAs was isolated, resolved on 15% of urea gels, and hybridized with probes complementary to the conserved region of mmu-mito-ncR-805. The MTHe membrane was stripped and reprobed with a U6-specific probe as a loading control. (B) The MLE12 cells were transfected with mmu-CR805 or non-targeting RNA and lysed, and the lysates were analyzed (total). Cytosolic fractions were obtained by separating crude mitochondria, which were further purified using Percoll gradient and treated with RNase I to remove non-mitochondrial RNAs. The RNAs were isolated from obtained fractions. Purity of the fractions was evaluated by comparing the relative expression levels of mtDNA-encoded mRNA of Rnr2, nucleus-encoded and localized Sno55-RNA, and cytosolic GAPDH mRNA with identical RNA inputs. [file Image_1.jpg]

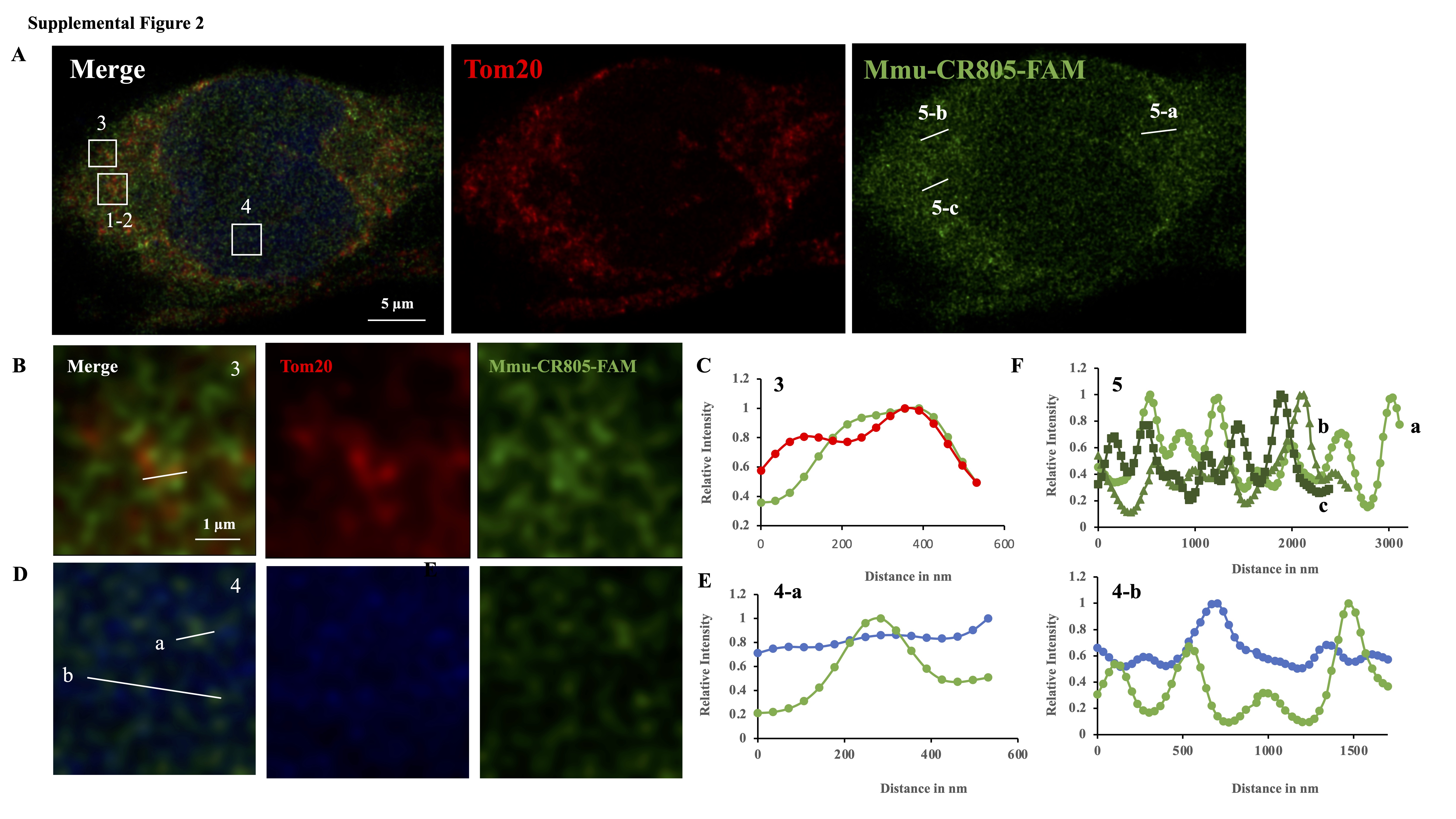

Supplement: Supplementary Figure 2 — The MLE12 cells were transfected with FAM-labeled mmu-CR805, fixed, and stained for Tom20. Images were acquired at a magnification of 100 times and zoom of 3.37 times on a Leica TCS SP8 microscope with accelerated deconvolution. (A) Single plane of representative cell images, with two boxed areas that contain Tom20-labeled structures (1–2 and 3) and one boxed area through the nucleus (4). (B) Enlarged area 3 from (A). (C) Graphs of relative red and green channel fluorescence intensity through the line-scanned region shown in (B). (D) Enlarged area 4. (E) Graphs of a relative blue and green channel fluorescence intensity through the line-scanned region shown in (D). (F) Line scans 5a–c were drawn to obtain the relative green channel intensity (FAM-labeled mmu-CR805) through cytoplasmic regions. [file Image_2.jpg]

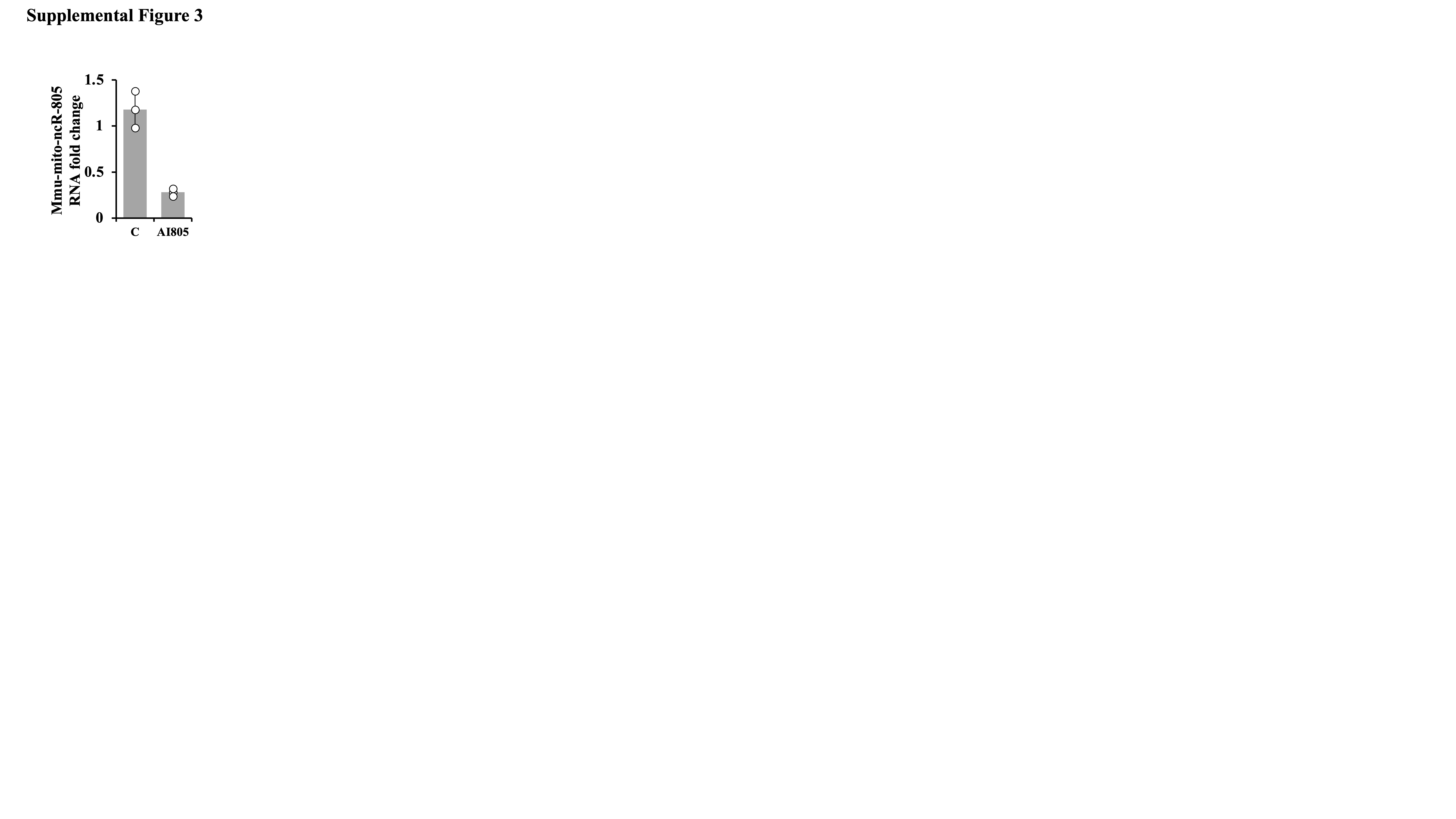

Supplement: Supplementary Figure 3 — Efficiency of inhibition by AI805. The MLE12 cells were transfected with AI805 or non-targeting RNA; 24 h post-transfection, the RNA was extracted and analyzed for the expression levels of mmu-CR805 containing transcripts. [file Image_3.jpg]
